# Prevalence of Cardiovascular Disease Risk Factors and NT pro-BNP for Cardiovascular Disease Risk Prediction among Older People Living with HIV in Almaty, Kazakhstan

**DOI:** 10.21203/rs.3.rs-9269044/v1

**Published:** 2026-04-15

**Authors:** Nursultan Nurzhigitov, Deborah Gustafson, Zhamilya Nugmanova, Alfiya Denebayeva, Aigerim Alimbekova, Gulnara Nugumanova, Gulmira Kalzhanbayeva, Ademi Sarsembiyeva, Jack DeHovitz

**Affiliations:** SPH, Kazakh National Medical University (KNMU); State University of New York Downstate Health Sciences University; SPH, Kazakh National Medical University (KNMU); Almaty AIDS Center; Almaty AIDS Center; SPH, Kazakh National Medical University (KNMU); SPH, Kazakh National Medical University (KNMU); SPH, Kazakh National Medical University (KNMU); State University of New York Downstate Health Sciences University

**Keywords:** HIV, aging, Cardiovascular disease, CVD risk, NT pro-BNP, Kazakhstan

## Abstract

**Introduction:**

Cardiovascular disease (CVD) is the leading cause of premature mortality in Kazakhstan and across Eastern Europe and Central Asia. As life expectancy increases among people living with HIV (PLWH) receiving antiretroviral therapy (ART), cardiovascular risk has become a major clinical concern. However, data on CVD risk distribution and cardiac biomarkers among older PLWH in Kazakhstan remain limited. This study aimed to estimate the prevalence of CVD risk factors, assess 10-year CVD risk using SCORE2/SCORE2-OP, and evaluate the role of NT-proBNP among PLWH aged ≥ 40 years in Almaty.

**Methods:**

A cross-sectional study was conducted among 150 PLWH aged ≥ 40 years attending the Almaty City AIDS Center between February and July 2023. Sociodemographic, behavioral, clinical, and laboratory data were collected. Ten-year CVD risk was calculated using SCORE2 (ages 40–69 years) and SCORE2-OP (≥ 70 years). Multivariable logistic regression was used to identify predictors of elevated NT-proBNP (≥ 125 pg/mL).

**Results:**

The mean age was 51.6 ± 10.4 years, and 54.7% of participants were male. ART coverage was 95%, with 76.7% achieving viral suppression. Smoking (76.1%) and alcohol use (65.5%) were highly prevalent. Overall, 29.3% of participants had low, 40.7% moderate, and 30.0% high 10-year CVD risk; 54% were classified as moderate or high risk. Elevated NT-proBNP (≥ 125 pg/mL) was observed in 36.3% of participants. In multivariable analysis, older age was independently associated with elevated NT-proBNP (adjusted odds ratio [aOR] per 1-year increase: 1.10; 95% CI: 1.04–1.15; p = 0.0004), whereas HIV viral load, blood pressure, sex, and triglyceride levels were not independently associated.

**Conclusions:**

More than half of older PLWH in this urban Kazakhstan cohort had moderate or high predicted 10-year CVD risk despite high ART coverage. Traditional cardiovascular risk factors were the primary drivers. Integration of routine CVD risk assessment and targeted prevention strategies into HIV care is urgently needed.

## INTRODUCTION

Non-communicable diseases (NCDs) account for approximately 84% of all deaths in Kazakhstan (KZ) (WHO, 2014a). In 2016, individuals in KZ had a 27% probability of dying before the age of 70 years from one of the four major NCDs—cardiovascular disease (CVD), diabetes, chronic respiratory disease, or cancer—with a substantially higher risk among men (37%) compared with women (19%) (WHO, 2017a). CVD remains the leading driver of premature mortality in KZ, particularly among men and individuals with high-risk behavioral profiles [1, 2].

Population-level data indicate that approximately 50% of adults in Kazakhstan were overweight or obese in 2020—one of the highest prevalences in the Eastern Europe and Central Asia (EECA) region [3]. Tobacco use is reported in 44% of men, alcohol consumption in 54%, and 25% of adults have hypertension [4, 5]. Additional risk factors include high salt intake, dyslipidemia, hyperglycemia, and physical inactivity. These patterns underscore the urgency of achieving Sustainable Development Goal target 3.4, which aims to reduce premature NCD mortality by one-third by 2030.

With the widespread availability of effective antiretroviral therapy (ART), life expectancy among people living with HIV (PLWH) increasingly approaches that of the general population. However, the epidemiology of age-related comorbidities among older PLWH in Kazakhstan remains largely uncharacterized. Delayed HIV diagnosis may result in prolonged exposure to systemic inflammation, immune activation, endothelial dysfunction, and metabolic dysregulation—mechanisms implicated in accelerated atherosclerosis among PLWH. [6]. Evidence from high-income countries demonstrates that older PLWH experience elevated prevalence of hypertension, diabetes, obesity, and dyslipidemia, even when virally suppressed [7, 8]. Data from low- and middle-income countries (LMICs), particularly in the EECA region, remain limited.

Approximately 40% of PLWH in KZ are aged ≥ 40 years [9, 10]. This population frequently experiences overlapping communicable and non-communicable disease risks, including high smoking prevalence, alcohol use, coinfections (e.g., hepatitis C virus), and socioeconomic disadvantages [11–15]. Among specific subgroups such as people who inject drugs, vascular risk may be further amplified due to infective endocarditis and chronic inflammatory burden [16–18]. Globally, PLWH have a 1.5–2-fold increased risk of myocardial infarction, heart failure, and other cardiovascular outcomes compared with HIV-negative individuals of similar age [19, 20].

Assessment of 10-year cardiovascular risk is recommended in routine HIV care. The European Society of Cardiology recently updated the SCORE2 (ages 40–69 years) and SCORE2-OP (ages ≥ 70 years) algorithms to estimate 10-year risk of fatal and non-fatal cardiovascular events. However, the performance and distribution of SCORE2/SCORE2-OP risk categories among PLWH in high-risk EECA settings have not been evaluated [21, 22].

In addition to traditional risk scores, biomarkers such as N-terminal pro-B-type natriuretic peptide (NTproBNP) have emerged as indicators of subclinical cardiac stress and predictors of cardiovascular morbidity and mortality [23]. Elevated NT-proBNP has been associated with cardiac dysfunction and may reflect early myocardial strain, including in PLWH [22, 23]. However, its distribution and potential clinical relevance in older PLWH in EECA settings are unknown.

To address these knowledge gaps, we conducted the Healthy Heart Study, a cross-sectional pilot investigation among PLWH aged ≥ 40 years in Almaty, Kazakhstan. The primary objective was to estimate the prevalence of major cardiovascular risk factors and characterize 10-year CVD risk distribution using SCORE2/SCORE2-OP algorithms. Secondary objectives were to evaluate NT-proBNP concentrations and examine associations with HIV-related parameters. To our knowledge, this is the first study in Kazakhstan and the EECA region to integrate standardized cardiovascular risk estimation and cardiac biomarker assessment within routine HIV care among older PLWH.

## METHODS

A cross-sectional study was conducted among a convenience sample of 150 PLWH ≥ 40y at the Almaty City AIDS Center (ACAC) in KZ over a ~ 6 month period (February–July 2023). Besides having HIV, inclusion criteria were being ≥ 40y, able to voluntarily complete all components of the study, resident of Almaty, and being a patient at the ACAC. Exclusion criteria included being < 40y, inability to voluntarily complete all components of the study, or COVID-19 or any COVID-19 symptoms within the past 90 days [24, 25]. There was modest time required for traveling to the ACAC. Participants were recruited consecutively during routine clinical visits due to feasibility constraints and the pilot nature of the study. The sample was not randomly selected and therefore may not fully represent all PLWH receiving care at the Almaty City AIDS Center or the broader population of PLWH in Kazakhstan.

Self-reported sociodemographic factors, clinical measures, CVD measures, HIV factors, and fast blood tests were measured in all participants. Sociodemographic factors included date of birth, sex at birth, gender, sexual orientation, partnership status (married, single, divorced, separated, cohabitating, widowed), type and years of employment/unemployment, maximum level of education attained. *Health behaviors* assessed included substance use, alcohol intake (type, intake frequency, and quantity), cigarette and other forms of smoking (number per day or per week), and sexual behaviors. Alcohol use disorder was assessed via the Alcohol Use Disorders Identification Test (AUDIT), and substance use disorder via the Drug Use Disorders Identification Test (DUDIT). Cognitive impairment assessed by Montreal Cognitive Assessment (MoCA). *Clinical assessments* included body weight in kilograms (kg), body height in meters, m), systolic and diastolic blood pressure in mmHg (SBP and DBP, respectively), and % blood oxygen saturation. Based on these assessments, vascular phenotypes included the following. Body mass index (BMI) was used to estimate overweight and obesity, as well as underweight. A healthy BMI was defined as 18.5–24.9 kg/m^2^, overweight as BMI 25–29.9 kg/m^2^, obesity as BMI > = 30kg/m^2^, and underweight as < 18.5 kg/m^2^ [26]. Normal oxygen saturation was defined as 95–100% and heartbeat as 60–90 beats per minute (bpm). Type 2 diabetes (T2D) was defined as a fasted HbA1c ≥ 6.5 or self-reported history of T2D [27]. Hypertension was defined as SBP ≥ 140 mmHg and/or DBP ≥ 90 mmHg or self-reported diagnosis [28]. CVD risk was calculated using two CVD risk scores composed of clinical and laboratory data. [29] SCORE2 and SCORE2-OP were created by the European Society of Cardiology to estimate standardized 10y CVD mortality rates. SCORE2 (< 70y) & SCORE2-OP (persons ≥ 70y) were developed using data from 445 prospective cohort studies in 13 countries in Western and Eastern Europe and the US. Seven factors are included: age, sex, smoking status, systolic blood pressure, total cholesterol and high-density lipoprotein (HDL) levels, and consideration of European CVD risk regions based on World Health Organization (WHO) data. Regional risk charts of predicted 10y cardiovascular disease risks by age group were initially classified as low CVD Risk (< 50y (< 2.5%); 50–69y (< 5%); ≥70y (< 7.5%)), moderate (< 50y (2.5%- <7.5%); 50–69y(5%- <10%); ≥70y (7.5%- <15%)), or high CVD Risk (< 50y (≥ 7.5%); 50–69y(≥ 10%); ≥70y (≥ 15%)) according to the European Society of Cardiology (ESC). For regression analysis, moderate and high risk categories were combined based on age-specific ESC thresholds for SCORE2 and SCORE2-OP. ART was assessed utilizing the Kazakh national clinical protocol for HIV treatment with two types of ART cocktails (preferable and alternative) approved by the Kazakhstan Ministry of Health. *Fasting blood samples* were collected before other clinical and survey measures. Biochemistry blood test was measured at the certified national MPK laboratory in KZ [30, 31]. Plasma levels of CD4 T cell count (CD4 count) and HIV viral load (HIV VL); and plasma levels of glucose, glycated hemoglobin (HbA1c), levels of total cholesterol, high density cholesterol (HDL), low density cholesterol (LDL), triglycerides, and hepatitis B virus (HBV), and hepatitis C virus (HCV) were measured. A low CD4 count was defined as < 200 cells/mm^3^ and an undetectable HIV VL as < 50 copies/ml. We also assessed serum levels of NT pro-BNP (ESC HF guidelines 2021). A higher level of NT pro-BNP was defined as ≥ 125 pg/ml. All surveys were administered in the Russian language. Study data were managed using the Centers for Disease Control and Prevention (CDC) KoBoToolbox platform (https://www.kobotoolbox.org/).

## Data Analysis

Descriptive Analysis: Our goal was to estimate the prevalence of CVD risk factors and indicators PLWH age > 40y in KZ with stratification by sex at birth and 10y age group. Risk factors and indicators included overweight and obesity; hypertension; T2D; high plasma levels of cholesterol and LDL; cigarette smoking; alcohol use; substance abuse; and levels of NP pro-BNP. We also associated 10-year CVD risk and indicators with HIV viral load and CD4 count. We characterized continuous variables (e.g., BMI, blood pressure) via computing means and standard deviations, and medians and interquartile ranges (IQR). Categorical variables were compared using the χ2 test. Analyses were stratified by sex and 10y age group. Results were considered significant at p < 0.05.

Multivariable Modeling: To identify factors associated with elevated NT-proBNP (≥ 125 pg/mL), multivariable logistic regression analysis was performed. Elevated NT-proBNP was defined according to European Society of Cardiology heart failure guidelines (≥ 125 pg/mL). Covariates were selected a priori based on clinical relevance and biological plausibility. Age (continuous), sex at birth, and diastolic blood pressure (continuous) were included as potential confounders. HIV-related variables included HIV viral load (detectable ≥ 50 copies/mL vs suppressed < 50 copies/mL), CD4 count (< 350 vs ≥ 350 cells/mm³), and duration of ART (years, continuous). Triglyceride levels (continuous) were included due to their borderline association in univariable analysis.

Adjusted odds ratios (aORs) with 95% confidence intervals (CI) were reported. Model diagnostics included assessment of multicollinearity using variance inflation factors (VIF < 5 considered acceptable) and evaluation of model fit using the Hosmer–Lemeshow goodness-of-fit test.

Missing data was < 5% for all variables; complete case analysis was performed. Sample Size Considerations: with a sample size of 150 participants, prevalence estimates around 50% have an approximate precision of ± 8% at the 95% confidence level. Given the pilot nature of the study, regression analyses were considered exploratory.

All statistical analyses were performed using R version 4.3.1 (R Foundation for Statistical Computing, Vienna, Austria). [32, 33].

## Ethical Approval

The study protocol obtained institutional review board (IRB) approval by the Kazakh National Medical University (Protocol No. 2022–23/21) Institutional Review Board. Written informed consent was obtained from each participant in accordance with the Declaration of Helsinki.

## Results

This cross-sectional study analyzed 150 PLWH (≥40 years) from the Almaty City AIDS Centre in 2023. The mean age was 51.6±10.4 years, and 54.7% were male. Five participants were aged >70 years, all of whom were male. Participants represented diverse socioeconomic backgrounds; 58% had at least secondary education and 60.7% owned their homes. Women were slightly younger and less likely to be employed, whereas men reported higher educational attainment. The overall ART coverage was 95%, with 76.7% achieving viral suppression (<50 copies/mL). Among all participants, 65.5% consumed alcohol; 76.1% were smokers and 26.5% drug users.

### Health Behavior and Lifestyle Factors

Behavioral data demonstrated a high prevalence of modifiable CVD risk factors among older PLWH. As shown in Table 1, smoking and alcohol use were more common among men, whereas physical inactivity did not differ significantly by sex.

Among those classified as high CVD risk, all were smokers; 87% of moderate-risk individuals smoked, versus <50% in the low-risk group.

### Clinical and Physiological Characteristics

Clinical assessment revealed a high prevalence of CVD abnormalities among older PLWH. As shown in Table 2, overweight and dyslipidemia were common, and elevated diastolic blood pressure was significantly associated with moderate to high cardiovascular risk.

### Cardiovascular Risk Estimation

**Figure 1:**
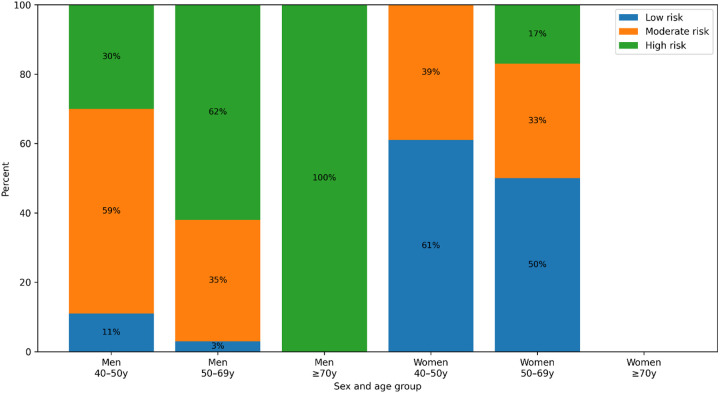
10-year cardiovascular disease risk using SCORE2 and SCORE2-OP among people living with HIV in Almaty, Kazakhstan, stratified by sex and age group.

10y CVD risk scores are shown in Figure 1. Low CVD risk was observed in 29.3% of participants; 40.7% had medium CVD risk; and 30% had high CVD risk. Among participants aged 40–49 years, 38% were classified as low risk, while 48% had moderate and 14% high CVD risk. Among those 50–69y, 46% had high CVD risk, 34% moderate and 20% low CVD risk. All participants at age ≥70y had high CVD risk and were men.

Associations between clinical variables and CVD risk category are presented in Table 3. An association between the initial DBP recorded and moderate to high CVD risk was observed. In addition, MoCA scores were associated with CVD risk. Median HIV VL was undetectable and median CD4 count was within the normal range among all CVD risk groups. There was a significant association between initial SBP, ART years, lipid levels, NT-proBNP and CVD risk. Behavioral factors, such as alcohol consumption, drug use, smoking were significantly associated with CVD risk. Participants with high CVD risk were 100% smokers, while in the moderate group, 87% reported smoking, and <50% of those in the low CVD risk reported smoking. Drug use prevalence ranged from 18.6% to 30.6% among all CVD risk groups.

Clinical characteristics differed across cardiovascular risk categories. As shown in Table 3, higher diastolic blood pressure, increased heart rate, and lower cognitive performance (MoCA score) were significantly associated with higher SCORE2/SCORE2-OP risk categories. No significant differences were observed for lipid parameters, glycemic control, HIV viral load, or CD4 count.

The 10-year cardiovascular disease (CVD) risk estimated by SCORE2/SCORE2-OP ranged from 1% to 38%, with a median of 5.5% (IQR: 2.5–9.5). Overall, 54% (n=81) of participants were classified as having moderate or high CVD risk (≥5%), while 46% (n=69) were categorized as low risk (<5%). Men had higher median risk values than women, and individuals aged ≥50 years were disproportionately represented in the moderate/high-risk group. As expected, age demonstrated the strongest correlation with SCORE %, given its central role in the algorithm, followed by systolic and diastolic blood pressure (ρ=0.53 and 0.52, respectively). NT-proBNP showed a weak positive correlation with SCORE percentage (ρ = 0.15) and showed a moderate correlation with age (ρ=0.34).

### NT-proBNP and Biochemical Profile

Elevated NT-proBNP (≥125 pg/mL) was observed in 36.3% of participants. Participants with elevated NTproBNP levels (≥125 pg/mL) demonstrated significantly higher HIV viral load (p=0.002) and borderline higher triglyceride levels (p=0.05) compared with those with lower NT-proBNP concentrations (<125 pg/ml). Although unadjusted analyses suggested an association between detectable HIV viral load and elevated NT-proBNP, this relationship did not persist after multivariable adjustment. No significant differences were observed for blood pressure, BMI, lipid fractions, glycemic control, or cognitive performance. Although NT-proBNP ≥125 pg/mL was more common among participants with elevated SCORE2/SCORE2-OP results, its association did not retain statistical significance after adjustment for age and sex, suggesting that biomarker elevation may partially reflect age-associated myocardial strain.

### Multivariable Analysis of Elevated NT-proBNP

In multivariable logistic regression analysis (see table 4), older age was independently associated with increased odds of elevated NT-proBNP (aOR per 1-year increase: 1.10; 95% CI 1.04–1.15; p=0.0004). Male sex (aOR 0.97; 95% CI 0.45–2.07; p=0.93), diastolic blood pressure (aOR per 1 mmHg increase: 0.97; 95% CI 0.92–1.01; p=0.15), detectable HIV viral load (aOR 1.61; 95% CI 0.72–3.60; p=0.25), and triglyceride levels (aOR per 1 mmol/L increase: 0.87; 95% CI 0.57–1.32; p=0.51) were not independently associated with NT-proBNP elevation after adjustment.

Model diagnostics demonstrated no evidence of multicollinearity, and goodness-of-fit testing indicated adequate model performance.

These findings indicate that NT-proBNP elevation in this cohort was primarily driven by age-related myocardial stress rather than HIV-specific or metabolic factors.

## Discussion

This study was conducted at a single urban HIV center using a convenience sample of clinic attendees. Although the Almaty City AIDS Center is one of the largest HIV treatment facilities in Kazakhstan, the sample may not be fully representative of all PLWH in Almaty or nationwide. Individuals not engaged in care, residing in rural areas, or with different socioeconomic characteristics may have different cardiovascular risk profiles. Therefore, findings should be interpreted as clinic-based estimates rather than nationally representative prevalence figures.

CVD among PLWH has become increasingly prevalent due to improved survival with ART, aging of the HIV population, and the growing burden of both communicable and non-communicable comorbidities. In this cross-sectional study of PLWH aged ≥40 years in Almaty, Kazakhstan, we evaluated traditional CVD risk factors, estimated 10-year CVD risk using SCORE2 and SCORE2-OP algorithms, and measured fasting NT-proBNP and metabolic biomarkers [34,35]. Consistent with Kazakhstan’s classification as a high CVD-risk region by the European Society of Cardiology (ESC), 54% of participants were categorized as having moderate or high predicted 10-year CVD risk. We used SCORE2 criteria as opposed to other criteria, because they were developed in Europe and therefore potentially more relevant for people in Kazakhstan.

CVD remains a leading cause of morbidity and mortality among PLWH [35]. The increasing burden of CVD in this population reflects not only extended life expectancy due to effective ART but also high prevalence of modifiable risk factors such as smoking, alcohol use, substance use, metabolic abnormalities, and possible long-term ART effects [36]. Current clinical guidelines recommend routine CVD risk assessment even in the absence of overt cardiovascular disease [37]. However, application of risk prediction tools developed in general populations requires caution, as the distribution and impact of risk factors may differ across regions and among PLWH. Regional epidemiology, including high background CVD mortality in Kazakhstan, supports the relevance of locally generated data to inform prevention strategies [38,39].

Beyond traditional risk factors, HIV-specific mechanisms may contribute to accelerated vascular aging. Chronic immune activation, systemic inflammation, endothelial dysfunction, and metabolic dysregulation are recognized pathways linking HIV infection to atherosclerosis and cardiovascular events. Although most participants in our cohort achieved viral suppression, the observed association between higher HIV viral load and elevated NT-proBNP suggests that uncontrolled viral replication may contribute to myocardial stress [40,41].

Risk assessment tools such as Framingham and earlier SCORE models have limitations when applied to PLWH [42,43]. The updated SCORE2 and SCORE2-OP algorithms incorporate contemporary data and regional CVD risk stratification, making them more appropriate for use in high-risk European regions such as Kazakhstan. In addition to estimated 10-year CVD risk, NT-proBNP represents a promising biomarker reflecting subclinical cardiac stress and myocardial strain [41,42]. Elevated NT-proBNP has been associated with heart failure, resistant hypertension, and cardiovascular mortality in both general and HIV-positive populations. In multivariable analysis, age emerged as the only independent factor associated with elevated NT-proBNP. Although higher HIV viral load was associated with NT-proBNP levels in unadjusted analyses, this association did not persist after adjustment for age and clinical factors. These findings suggest that NT-proBNP elevation in this cohort primarily reflects age-related myocardial strain rather than HIV-specific mechanisms.

These findings underscore the need to integrate comprehensive cardiovascular risk management into routine HIV care. In addition to ART optimization and viral suppression, interventions targeting smoking cessation, blood pressure control, lipid management, and lifestyle modification are critical. Incorporating biomarker assessment, such as NT-proBNP measurement among higher-risk individuals, may further enhance prevention strategies, although prospective validation is required.

This study has limitations. The cross-sectional design precludes causal inference, and the convenience sample was drawn from a single urban HIV center, which may limit generalizability to other regions of Kazakhstan. Additionally, modest sample size may have reduced power to detect independent associations for certain biomarkers. Nevertheless, this investigation represents the first integrated assessment of standardized CVD risk estimation and NT-proBNP measurement among older PLWH in Kazakhstan.

Overall, our findings highlight the urgent need to integrate comprehensive cardiovascular risk assessment and prevention strategies into routine HIV care. Beyond maintaining viral suppression, clinical management should prioritize smoking cessation, blood pressure control, lipid management, and lifestyle modification.

## Conclusion

This is the first cross-sectional study in Kazakhstan to comprehensively characterize cardiovascular disease (CVD) risk among people living with HIV (PLWH) aged ≥40 years. More than half of participants had moderate or high predicted 10-year CVD risk despite high antiretroviral therapy (ART) coverage and viral suppression. Older age, male sex, smoking, and elevated diastolic blood pressure were strongly associated with higher predicted cardiovascular risk.

Elevated NT-proBNP levels were observed in more than one-third of participants and were strongly associated with increasing age. After adjustment, HIV-related and metabolic factors were not independently associated with NT-proBNP elevation, suggesting that biomarker levels primarily reflect age-related myocardial stress in this cohort. Although NT-proBNP was not independently associated with predicted CVD risk after adjustment, its high prevalence highlights the need for further investigation of its role in cardiovascular risk stratification among PLWH.

These findings highlight the potential importance of integrating cardiovascular risk assessment into HIV care settings similar to the Almaty City AIDS Center and warrant further multi-center, nationally representative studies.

## Figures and Tables

**Table 1–:** Behavioral data reveal extensive exposure to modifiable CVD risks

Behavior	Overall %	Male *%*	Female %	p Value
Current smoker	76.1	91.4	64.7	<0.001
Alcohol (past month)	65.5	70.1	50.3	0.009
Drug use (ever)	26.5	25.6	8.8	0.038
Physical inactivity	37.2	31.5	43.1	0.12

**Table 2 –:** Clinical Parameters

Parameter	Mean ± SD	Abnormal %
BMI (kg/m^2^)	27.1±5.3	38% overweight
SBP (mmHg)	120.0±10.0	–
DBP (mmHg)	79.1±10.0	31% ≥ 90 mmHg
Heart Rate (bpm)*	74±8.2	–
Hypertension (≥140/90)	31.0	–
Fasting glucose (mmol/L)	5.9±1.2	24% ≥6.1
Total Cholesterol (mmol/L)	5.3±1.1	43% dyslipidaemic

Bpm: beats per minutes

**Table 3 –:** Association Between Cardiovascular Risk and Clinical Variables among PLWH in the Healthy Heart Study using SCORE2 and SCORE2-OP

*Clinical Variables*	Low CVD Risk	Moderate CVD Risk	High CVD Risk	*p value*
	*Median (1^st^ Qu. – 3d Qu.)*	*Median (1^st^ Qu–3d Qu.)*	*Median (1^st^ Qu–3d Qu.)*	
Age (y)	45.0 (42.5–50.0)	48.0 (46.0–53.0)	56.0 (51.0–63.0)	0.001
BMI (kg/m^2^)	22.3(21.1–25.3)	24.4 (21.1–27.0)	23.2 (21.6–26.3)	0.146
SBP(mmHg)	110.0 (110.0–120.0)	120.0 (120.0–130.0)	130.0 (120.0–130.0)	0.067
DBP(mmHg)	70.0 (70.0–80.0)	80.0 (70.0– 80.0)	85.0 (80.0–90.0)	0.013
Heartbeat(bpm)	78.0 (72.0–86.0)	80.0 (76.0–86.0)	82.0 (70.0–82.0)	0.047
ART years	5.0 (2.5–7.0)	5.5 (3.0–9.0)	8.0 (4.0–11.0)	0.591
MoCA	24.0 (23.0–26.0)	23.0 (22.2–25.0)	22.0 (21.0–24.0)	<0.001
HbA1C (%)	5.5 (5.2–5.8)	5.6 (5.3–5.8)	5.6 (5.4–5.9)	0.942
LDL (mmol/l)	2.4 (2.1–2.9)	2.8 (2.1–3.3)	2.9 (2.2–3.7)	0.343
HDL (mmol/l)	1.4 (1.1–1.6)	1.2 (1.0–1.4)	1.1 (1.0–1.4)	0.331
NT-proBNP (pg/ml)	90.0 (55.9–161.8)	66.5 (42.2–168.7)	132.1 (64.0–279.3)	0.112
HIV VL (copies/ml)	<50 (<50)	<50 (<50)	<50 (<50–66)	0.170
CD4 count/ml^3^	428.0 (262.5–607.0)	438.0 (331.0–645.2)	437.0 (275.0–590.0)	0.643

Notes: *BMI – Body mass index. SBP – systolic blood pressure. DBP – diastolic blood pressure; heartbeat per minute(bpm). MoCA – Montreal Cognitive Assessment. ART – years, period of receiving Antiretroviral therapy in years. HbA1C – A hemoglobin A1C. HDL – High density cholesterol; LDL, Low density cholesterol. HIV VL – HIV viral load. CD4, cluster of differentiation. Continuous variables are presented as median (interquartile range). Group comparisons across cardiovascular risk categories were performed using the Kruskal–Wallis test. p values <0.05 were considered statistically significant*

**Table 4 –:** Multivariable logistic regression predicting elevated NT-proBNP (≥125 pg/mL)

Predictor	aOR	95% CI	p-value
Age (per 1 year)	1.10	1.04–1.15	0.0004
Male sex	0.97	0.45–2.07	0.93
DBP (per 1 mmHg)	0.97	0.92–1.01	0.15
Detectable HIV VL (>50 copies/mL)	1.61	0.72–3.60	0.25
Triglycerides (per 1 mmol/L)	0.87	0.57–1.32	0.51

## Data Availability

Original data collected within this study is not publicly available, as it might contain sensitive information. De-identified data can be shared based on a reasonable request by sending an email to nurzhigitov.nursultan@gmail.com.

## Key Message

Scientific knowledge on the HIV/noncommunicable disease intersection, especially related to cardiovascular disease risk and outcomes in people living with HIV in Kazakhstan, is important related to increasing numbers of older people living with HIV. This study adds first-ever reported data on people with HIV who are 40 years and older in Kazakhstan. Findings from this study will guide intervention and prevention strategies and guidelines among people living with HIV in Kazakhstan and other low- and middle-income countries.
